# Supplementary material for: Overseas background executives and enterprise ESG performance -The moderating effect of the executive pay gap
Source: PLoS One. 2025 May 28;20(5):e0324645. doi: 10.1371/journal.pone.0324645 (PMC12118867; doi:10.1371/journal.pone.0324645)
Supplement: Attachment — Submitted filename: Response to Reviewers.docx [file pone.0324645.s002.docx]

**Revision Notes**

**Dear Respected Editor and Reviewers,**

Thanks for your letter and for the reviewers’ kind comments concerning our manuscript entitled “Overseas background executives and enterprise ESG performance - the moderating effect of the executive pay gap” (ID: PONE-D-24-50043).

Those comments are all valuable and helpful for us to improve our paper, as well as the important guiding significance to our research. We have studied the comments carefully and have made substantial revisions, which we hope will meet with approval. Revised portions are marked in red on the paper. The main corrections in the paper and responses to reviewers’ comments are described below. Meanwhile, we also invite Kassi Diby Francois Noel Koffi, a native speaker and foreign postdoctoral fellow at Henan University, to help edit the language of this paper. Furthermore, we collaborate with Shenyang Hai, a returned overseas Chinese, Hainan Provincial Category E High-Level Talent, PhD Supervisor at Hainan University, to enhance the language quality of this manuscript.

Thanks again for your time and letter.

Yours Sincerely

Baoping Liu

Editor/Associate Editor

**Comment 1:** Please ensure that your manuscript meets PLOS ONE's style requirements, including those for file naming.

**Response: We have carefully read the style requirements of the journal, including the naming requirements, according to the website provided by the editor. We have made corrections to the requirements.**

**Comment 2:** Please state what role the funders took in the study.

**Response: This work is supported by the Major Program Grants for Applied Research from the Education Department of Henan Province (2023-YYZD-04), China. The funders had no role in study design, data collection and analysis, decision to publish, or preparation of the manuscript.**

**Comment 3:** Please upload the minimal anonymized data set necessary to replicate your study findings to a stable, public repository and provide us with the relevant URLs, DOIs, or accession numbers.

**Response: Thanks for the editor’s reminder. We have uploaded the relevant research data as Supporting Information files.**

**Comment 4:** Your abstract cannot contain citations. Please only include citations in the body text of the manuscript, and ensure that they remain in ascending numerical order on first mention.

**Response: Thank you so much. We have carefully examined the abstract and manuscript sections of this paper and have confirmed that there are no citations in the abstract section. The citations in the main text of the manuscript are indicated as ascending numbers when first mentioned.**

**Reviewer 1.**

**Comment 1:** A recent study from Liu et al. (2024) have examined the impact of the Chinese CEOs with foreign experience on the firm's ESG performance. Please justify the differences. Additionally, many studies have examined the connection between Executive Compensation and ESG Performance, as detailed in the reference section.

The authors need to identify and articulate what sets their study apart from previous research. I recommend the authors can try to explore additional dimensions of CEO overseas experience (e.g., duration, type of overseas exposure, or specific skills gained) instead of using a binary dummy variable. This could provide deeper insights into its impact on ESG performance. Besides, the authors may consider exploring the mechanisms through which overseas executives influence ESG performance.

**Response: We would like to express our sincere gratitude to the reviewers for their valuable suggestions and comments. After carefully analyzing the feedback, we have identified that this comment primarily encompasses three key aspects. Accordingly, we will address each of these aspects individually in our response.**

**(1) The reviewer noted that existing studies have examined the impact of Chinese CEOs with overseas experience on corporate ESG performance, which we cited and compared in the second paragraph of the first section of our paper. Below, we specify the key differences between our study and theirs:**

**First, distinction in the scope of “executives” versus “CEOs”. Our research focuses on senior executives with overseas backgrounds, which extends beyond CEOs to include other high-level overseas personnel within the firm. Specifically, our definition of “executives” aligns with the China Stock Market & Accounting Research (CSMAR) database, encompassing not only CEOs but also senior management roles disclosed in corporate annual reports, such as Deputy General Managers, Vice Presidents, Chief Accountants, Chief Economists, Chief Engineers, and equivalent positions.**

**Second, theoretical divergence. While Liu et al. (2024) employed imprinting theory, stakeholder theory, and resource dependence theory, our study is grounded in Upper Echelons Theory and Tournament Theory to underpin the research framework.**

**Third, differences in data timelines. The study mentioned by the reviewer covers the period 2011–2020, whereas our dataset spans 2008–2022, providing a more comprehensive temporal analysis of the relationship between overseas executives and ESG performance.**

**Fourth，variation in moderating variables. The prior study utilized moderators such as female CEOs, CEO educational attainment, and state-owned enterprises. In contrast, we concentrate on the moderating role of executive pay gaps, which we further categorize into internal pay gaps (disparities among executives within the firm) and external pay gaps (disparities relative to industry peers) for granular investigation.**

**Therefore, we made changes in the revision of this paper as follows. A preliminary literature search reveals that only Liu et al. have indicated that Chinese CEOs’ overseas experience improves corporate ESG performance. However, the CEO is only one member of the corporate executive team, which differs from the focus of this study. According to Upper Echelons Theory, the background and experiences of top executives may influence corporate decision-making by shaping their perceptions and interpretations of external environments. Therefore, there remains a research gap regarding the impact of overseas background executives on corporate ESG performance.**

**Two new references are cited in the text.**

1. Liu Y, Zhang F, Zhang H. CEO foreign experience and corporate environmental, social, and governance (ESG) performance. Bus Strateg Environ. 2023;33(4):3331-55. https://doi.org/10.1002/bse.3647

2. Hambrick DC. Upper echelons theory: An update. Acad Manage Rev. 2007;32(2):334-43. https://doi.org/10.5465/AMR.2007.24345254

**(2) We acknowledge that numerous studies have explored the relationship between executive compensation and ESG performance, and we have cited the key references provided by the reviewer in the first section of our paper. However, our study differs by delving deeper into executive compensation, proposing that internal pay gaps (disparities among executives within the firm) and external pay gaps (disparities relative to industry peers) may play a moderating role in the relationship between overseas-experienced executives and corporate ESG performance. Thus, our research contributes distinctively by refining and expanding the understanding of how executive compensation structures interact with overseas backgrounds to shape ESG outcomes.**

**Therefore, we made changes in the revision of this paper as follows. Additionally, the issue of pay gaps has attracted widespread societal attention, which underscores the potential moderating role of executive pay gaps in the relationship between overseas background executives and corporate ESG performance. Cohen et al. note that incorporating ESG metrics into executive compensation contracts has become an internationally prevalent practice. Meng et al. believe that linking executive pay to ESG performance can enhance ESG outcomes, while ESG practices, in turn, may increase executive compensation. Fu et al. further reveal that a greater number of overseas background executives correlates with larger internal pay disparities within firms. According to tournament theory, compensation gaps among executives participating in promotion tournaments can serve as effective incentives. Widening pay disparities may motivate executives to work harder, reduce agency costs, and improve corporate performance.**

**The above red paragraph is the part of this section of the article, in which two new references are cited, as shown below.**

1. Cohen S, Kadach I, Ormazabal G, Reichelstein S. Executive Compensation Tied to ESG Performance: International Evidence. J Account Res. 2023;61(3):805-53. https://doi.org/10.1111/1475-679x.12481

2. Meng T, Lu D, Yu D, Yahya MH, Zariyawati MA. Is executive compensation	aligned with the company’s ESG objectives? Evidence from Chinese listed companies	based on the PSM-DID approach. Hum Soc Sci Commun. 2024;11(1):1-15. https://doi.org/10.1057/s41599-024-04094-y

**(3) We sincerely appreciate the reviewer’s valuable suggestions to explore alternative measures of overseas background executives and to investigate the mechanisms through which such executives influence ESG performance. These are excellent recommendations, and we are deeply grateful for them. In our study, during the formulation of hypotheses, empirical testing, and robustness checks, we employed five distinct measures of overseas-experienced executives: a binary variable (presence/absence), the proportion of executives with overseas experience within the leadership team, the count of overseas-experienced executives, and the presence of overseas background CEOs or board chairs. Future research could explore additional dimensions of overseas experiences, such as their duration, types of overseas exposure (e.g., academic, professional, or cultural), or specific skills acquired abroad.**

**Additionally, while conducting theoretical analysis, hypothesis development, and empirical testing, we did not identify a definitive mechanism explaining how overseas background executives influence corporate ESG performance. This constitutes a limitation of our study and a promising direction for future research. We sincerely thank the reviewer for raising this critical point, which will guide our ongoing efforts to deepen understanding in this area.**

**Comment 2:** While the significance of overseas executives is highlighted, it would help to specify why this focus is particularly relevant to China’s current stage of economic development.

**Response: Thanks for the reviewer’s suggestion. We have revised the first paragraph of the introductory section to explicitly highlight the significance of overseas background executives in China’s current economic context, ensuring that our approach aligns with the relevant requirements. The revised text reads as follows:**

**With the deepening implementation of the talent-driven development strategy, China’s economy has transitioned from a phase of rapid growth to a stage of high-quality development. Under the new era of economic globalization and continuous enhancement of comprehensive national strength, China has witnessed a remarkable “reverse flow of talent” in recent years, with over a million high-level overseas professionals choosing to return for domestic development. A significant proportion of these returnees have assumed core managerial positions in local enterprises. These versatile talents with international perspectives, global resources, and transnational networks have not only introduced advanced governance philosophies and technical expertise from abroad, but also played a pivotal bridging role in driving national industrial upgrading and stimulating corporate innovation. They constitute indispensable strategic resources for China’s economic transformation and social reform. In practical terms, while enterprises require substantial high-level talent to ensure sustainable development, the imperative for sustainability simultaneously demands that corporations establish ESG systems and strengthen ESG management. This dual requirement conversely compels businesses to employ more specialized technical experts and senior executives.**

**Two new references are cited in the text.**

1. Zhang C, Fu P. Overseas-returned executives and their roles in firm performance: evidence from China. Asia-Pac J Account E. 2022;29(3):622-31. https://doi.org/10.1080/16081625.2020.1754257

2. Xu Z, Hou J. CEO overseas experience, dynamic capabilities and corporate digital transformation: An imprinting theory perspective. Hum Soc Sci Commun. 2024;11(1):1-14. https://doi.org/10.1057/s41599-024-03227-7

**Comment 3:** While stakeholder theory, top echelon theory, and imprinting theory are presented, their integration feels somewhat fragmented. The narrative jumps between theories without clearly demonstrating how they build upon one another to explain the observed phenomena.

**Response:** **Thank you so much. In the original manuscript, we did indeed encounter the issue highlighted by the reviewer regarding the application of theoretical frameworks. After further reflection, we have revised the manuscript to remove references to Stakeholder Theory and Imprinting Theory. Instead, we now focus solely on Upper Echelons Theory to explain how the educational and professional backgrounds of senior executives reshape their values and, consequently, influence their strategic decision-making. The revised text can be found in the second paragraph of Section 1 in Part 2 of the article, as detailed below:**

**According to the Upper Echelons Theory, the educational and professional experiences of executive team members reshape their values, thereby influencing their strategic choices. Executives with overseas backgrounds—those who have studied or worked abroad—are influenced by the values and cognitive frameworks of foreign countries and regions, which further shape their strategic decision-making behaviors. Giannetti et al. found that overseas background executives apply advanced technologies and practices learned abroad to domestic firms, thereby enhancing corporate performance. In China, executives with overseas backgrounds primarily come from Western developed countries. They integrate knowledge from ESG frameworks in Europe and the U.S., and possess robust theoretical and practical foundations in ESG, which enables them to formulate and implement ESG strategies, ultimately improving corporate ESG performance. Therefore, overseas background executives are influenced by the ESG system and culture of developed countries during the critical period of growth. Their risk preferences, decision-making inclinations, and final choices will enhance the effectiveness and scientific rigor of ESG strategies. Additionally, given the limitations of individual knowledge structures and cognitive dimensions, the proportion of overseas background executives within the leadership team also plays a critical role in corporate ESG performance.**

**Comment 4:** When the authors consider the impact of specific years, they select the 2015 China financial crisis and the 2020 COVID-19 pandemic as two key events for the Year Impact Test, focusing on their relevance to the high-quality development of enterprises. However, why is the 2008 global financial crisis not included in this analysis?

**Response: We adopted a year-specific shock test in our study, which examines the impact of major events occurring in selected years. As rightly noted by the reviewer, the 2008 financial crisis should indeed be considered. However, since our dataset does not include pre-2008 data and the post-2008 results have already been validated, no control group is available to isolate the crisis’s effects. We acknowledge that for event-specific shocks (e.g., crises or policy changes), an alternative approach would involve excluding data from specific periods (e.g., 2008–2010, 2015–2017, or 2020–2022) for empirical testing, which differs from our current year-focused shock methodology. This distinction is clarified in the revised first paragraph of Section 4 in Part 5 of the article, where we have rewritten the relevant explanation as follows:**

**The empirical analysis in this study utilizes a sample spanning 2008–2022, an extended timeframe during which results may be influenced by policy shifts or socio-economic events relevant to the research focus. Notable examples include the 2008 global financial crisis, the 2015 Chinese stock market crash, and the 2020 COVID-19 pandemic—all of which imposed new demands on corporate sustainable development and significantly impacted ESG performance, potentially introducing bias to baseline regression conclusions. However, given that 2008 marks the starting year of our dataset and pre-2008 data is unavailable for comparison, the 2008 global financial crisis is excluded from the event study analysis. Instead, we focus on the 2015 financial turbulence and the 2020 COVID-19 pandemic outbreak, which disrupted global socio-economic systems.**

**Comment 5:** The study found that the influence of overseas executives on ESG performance is more pronounced in state- owned enterprises (SOEs), large-scale firms, firms in the eastern region, and those in high-pollution industries. However, the analysis section lacks further explanation and provides only a general statistical description.

**Response: The reviewer suggested that in the heterogeneity analysis section of the paper, in addition to describing the more pronounced results for state-owned enterprises (SOEs), large-scale firms, enterprises in eastern regions, and high-pollution industries, explanations for these patterns should also be provided. In response, we have incorporated causal interpretations into the revised manuscript. These additions are detailed in the heterogeneity analysis section (Section 6) of the article.**

**6.2.1 Analysis of ownership heterogeneity**

**The impact of overseas background executives on corporate ESG performance may differ between state-owned enterprises (SOEs) and non-SOEs due to inherent variations in their governance structures and strategic priorities. To examine this heterogeneity, we construct a dummy variable (SOE) where SOE = 1 for state-owned firms and SOE = 0 for non-state-owned firms. Regression results from Columns (1) and (2) of Table 12 reveal that the interaction terms *Oversea1*SOE* and *Oversea2*SOE* yield coefficients of 1.890 and 6.759, respectively, both statistically significant at the 1% level. This indicates that overseas-experienced executives enhance ESG performance more effectively in SOEs compared to non-SOEs.**

**A plausible explanation is that State-owned enterprises (SOEs) possess significant scale advantages and economic strength, granting them greater market competitiveness and bargaining power. Moreover, they bear heightened economic, political, and social responsibilities compared to non-SOEs. Consequently, the institutional environment of SOEs provides stronger incentives and structural support for overseas background executives to actively fulfill social responsibilities, engage in environmental governance, and implement decisions that enhance corporate ESG performance ratings.**

**6.2.2 Analysis of scale heterogeneity**

**Differences in firm size lead to variations in capital accumulation, green innovation capabilities, and sustainability management systems, which may result in heterogeneous effects of overseas-experienced executives on ESG performance. To examine this, we categorize firms into two groups based on the annual median of firm size (Scale): firms above the median are assigned Scale = 1, and those below Scale = 0. The re-estimated results of Model (1) are shown in Columns (3) and (4) of Table 12. It can be seen that the interaction terms *Oversea1*Scale* and *Oversea2*Scale* yield coefficients of 2.416 and 6.044, respectively, both statistically significant at the 1% level. This indicates that overseas-experienced executives exert a stronger ESG-enhancing effect in larger firms compared to smaller counterparts.**

**A plausible explanation is that larger firms typically possess greater capabilities and advantages in R&D innovation, resource integration, risk resilience, and strategic planning. These strengths enable overseas-experienced executives to leverage enhanced motivation and institutional resources to improve corporate ESG performance.**

**6.2.3 Analysis of regional heterogeneity**

**Regional disparities in development levels may lead to heterogeneous effects of overseas background executives on corporate ESG performance. To investigate this, we categorize the sample into Eastern region and Central-Western region groups based on national policies and provincial economic development levels, re-estimating Model (1). Results are presented in Columns (1) and (2) of Table 13. we find that the interaction terms *Oversea1*East* and *Oversea2*East* yield coefficients of 1.286 and 3.659, respectively, both statistically significant at the 1% level. This indicates that overseas background executives exert a stronger ESG-enhancing effect in the economically advanced Eastern region compared to the less-developed Central and Western regions.**

**Maybe because the Eastern region’s more mature market mechanisms and lower governmental intervention create an environment where firms face heightened demands for sustainability from governments, society, and investors. To gain a competitive edge, overseas background executives in this region are incentivized to prioritize ESG improvements, leveraging their expertise to align corporate strategies with market expectations.**

**6.2.4 Analysis of industry heterogeneity**

**The motivations and objectives for adopting ESG strategies differ significantly between firms in heavily polluting industries and those in other sectors. To explore this heterogeneity, we divide the sample into two groups: firms in heavily polluting industries and those in non-polluting industries, re-estimating Model (1). Results are presented in Columns (3) and (4) of Table 13. We find that the interaction terms *Oversea1*Pollute* and *Oversea2*Pollute* yield coefficients of 1.627 and 4.765, respectively, are statistically significant at the 1% level. This indicates that overseas background executives exert a more pronounced ESG-enhancing effect in heavily polluting industries compared to other sectors.**

**This may be because, in the reality of advocating green development, firms in heavily polluting industries face greater cost pressures and environmental regulatory pressures. To comply with national and local environmental regulations, protect the environment, and promote sustainable development, these firms have stronger incentives to improve ESG performance. Overseas-experienced executives, leveraging their expertise in global sustainability practices, play a critical role in driving such improvements, aligning corporate operations with stricter environmental and social governance standards.**

**Comment 6:** It is recommended that the study’s limitations be included at the end of the study.

**Response: Thank you for the recommendation to include the research limitations in the concluding section of the study. We greatly appreciate your suggestion. In the revised manuscript, we have explicitly addressed the limitations of this research within the Conclusion section, as detailed below:**

**7.3 Limitations and future research**

**Despite the valuable insights provided by this study, there are still some limitations. First, while this study focuses on executives’ overseas backgrounds, it does not provide a granular analysis of these backgrounds—such as the countries of origin, duration of overseas exposure, or hierarchical authority of the executives. Additionally, other leadership characteristics (e.g., academic training and financial expertise) may shape decision-making and thereby influence corporate ESG performance. Future research should conduct detailed examinations of these dimensions to deepen understanding of how overseas experiences interact with other attributes to drive sustainability outcomes.**

**Second, while this study examines corporate ESG performance through the lens of executives with overseas backgrounds, numerous other internal factors (e.g., board diversity, innovation incentives) and external determinants (e.g., regulatory pressures, stakeholder activism) remain underexplored. Future studies could broaden the scope by systematically investigating these additional dimensions to map the drivers of ESG outcomes.**

**Third, while this study examines the impact of overseas-experienced executives on corporate ESG performance, it lacks an in-depth analysis of the underlying mechanisms. Future research should prioritize exploring the pathways through which such executives influence ESG outcomes, thereby refining and expanding the current body of knowledge.**

**Reviewer 2.**

**Comment 1:** The first paragraph of the introduction should be rewritten, please focus on the research topic.

**Response: We sincerely appreciate the reviewer’s suggestion. In the revised manuscript, we have restructured the first paragraph of the Introduction to sharpen the focus on the research theme and enhance its alignment with the study’s core objectives. The updated text is as follows:**

**With the deepening implementation of the talent-driven development strategy, China’s economy has transitioned from a phase of rapid growth to a stage of high-quality development. Under the new era of economic globalization and continuous enhancement of comprehensive national strength, China has witnessed a remarkable “reverse flow of talent” in recent years, with over a million high-level overseas professionals choosing to return for domestic development. A significant proportion of these returnees have assumed core managerial positions in local enterprises. These versatile talents with international perspectives, global resources, and transnational networks have not only introduced advanced governance philosophies and technical expertise from abroad, but also played a pivotal bridging role in driving national industrial upgrading and stimulating corporate innovation. They constitute indispensable strategic resources for China’s economic transformation and social reform. In practical terms, while enterprises require substantial high-level talent to ensure sustainable development, the imperative for sustainability simultaneously demands that corporations establish ESG systems and strengthen ESG management. This dual requirement conversely compels businesses to employ more specialized technical experts and senior executives. Given the complex interplay between corporate leadership and sustainable development, coupled with growing attention from investors and financiers to environmental, social, and governance (ESG) standards, it becomes critically important to thoroughly investigate whether and how executives with overseas backgrounds influence corporate ESG performance.**

**Four new references are cited in the text.**

1. Zhang C, Fu P. Overseas-returned executives and their roles in firm performance: evidence from China. Asia-Pac J Account E. 2022;29(3):622-31. https://doi.org/10.1080/16081625.2020.1754257

2. Xu Z, Hou J. CEO overseas experience, dynamic capabilities and corporate digital transformation: An imprinting theory perspective. Hum Soc Sci Commun. 2024;11(1):1-14. https://doi.org/10.1057/s41599-024-03227-7

3. Qing L, Li P, Dagestani AA, Woo C, Zhong K. Does climate change exposure impact on corporate finance and energy performance? Unraveling the moderating role of CEOs’ green experience. J Clean Prod. 2024; 461:142653. https://doi.org/10.1016/j.jclepro.2024.142653

4. Qing L, Dagestani AA, Nam E, Wang C. Does firm-level exposure to climate change influence inward foreign direct investment? Revealing the moderating role of ESG performance. Corp Soc Resp Env Ma. 2024;31(6):6167-83. https://doi.org/10.1002/csr.2917

**Comment 2:** The Introduction needs to be rearranged, and more work is needed to strengthen the theoretical basis. Additionally, how this study bridges the research gaps should be elucidated.

**Response: We sincerely thank the reviewer for the valuable suggestions. In the revised manuscript, we have incorporated the recommended articles provided by the reviewer and reorganized the Introduction section in accordance with the suggested structure and formatting guidelines. The specific revisions are outlined below:**

**With the deepening implementation of the talent-driven development strategy, China’s economy has transitioned from a phase of rapid growth to a stage of high-quality development. Under the new era of economic globalization and continuous enhancement of comprehensive national strength, China has witnessed a remarkable “reverse flow of talent” in recent years, with over a million high-level overseas professionals choosing to return for domestic development. A significant proportion of these returnees have assumed core managerial positions in local enterprises. These versatile talents with international perspectives, global resources, and transnational networks have not only introduced advanced governance philosophies and technical expertise from abroad, but also played a pivotal bridging role in driving national industrial upgrading and stimulating corporate innovation. They constitute indispensable strategic resources for China’s economic transformation and social reform. In practical terms, while enterprises require substantial high-level talent to ensure sustainable development, the imperative for sustainability simultaneously demands that corporations establish ESG systems and strengthen ESG management. This dual requirement conversely compels businesses to employ more specialized technical experts and senior executives. Given the complex interplay between corporate leadership and sustainable development, coupled with growing attention from investors and financiers to environmental, social, and governance (ESG) standards, it becomes critically important to thoroughly investigate whether and how executives with overseas backgrounds influence corporate ESG performance.**

**Existing research has shown that executives with overseas backgrounds can enhance corporate R&D investment and the number of patent applications, effectively improve corporate operations and boost corporate performance, strengthen corporate risk-taking and enterprise performance, and further promote green innovation. Moreover, academic studies on the factors that influence corporate ESG performance can be categorized into macro and micro levels. At the macro level, factors such as national economic development levels, regional cultural differences, changes in party and government leadership in cities, government green financial policies, sustainable public procurement, and market competition shocks can influence corporate ESG performance. At the micro level, corporate ESG performance is also affected by factors such as firm size, performance feedback, online attention, managerial myopia, and board size and diversity. Overall, current domestic and international research lacks detailed studies on the relationship between executives with overseas backgrounds and corporate ESG performance. A preliminary literature search reveals that only Liu et al. have indicated that Chinese CEOs’ overseas experience improves corporate ESG performance. However, the CEO is only one member of the corporate executive team, which differs from the focus of this study. According to Upper Echelons Theory, the background and experiences of top executives may influence corporate decision-making by shaping their perceptions and interpretations of external environments. Therefore, there remains a research gap regarding the impact of overseas background executives on corporate ESG performance, and this gap motivates the first key research question of our study:**

**RQ1. Can executives with overseas backgrounds influence corporate ESG performance?**

**Additionally, the issue of pay gaps has attracted widespread societal attention, which underscores the potential moderating role of executive pay gaps in the relationship between overseas background executives and corporate ESG performance. Cohen et al. note that incorporating ESG metrics into executive compensation contracts has become an internationally prevalent practice. Meng et al. believe that linking executive pay to ESG performance can enhance ESG outcomes, while ESG practices, in turn, may increase executive compensation. Fu et al. further reveal that a greater number of overseas background executives correlates with larger internal pay disparities within firms. According to tournament theory, compensation gaps among executives participating in promotion tournaments can serve as effective incentives. Widening pay disparities may motivate executives to work harder, reduce agency costs, and improve corporate performance. Building on this analysis, this paper categorizes executive pay gaps into internal and external disparities to address our second research question:**

**RQ2. Do executive pay gaps moderate the impact of overseas background executives on corporate ESG performance?**

**To address the issues mentioned above, this study utilizes panel data from China’s Shanghai and Shenzhen A-share listed companies spanning 2008–2022 as the research sample. We employ a two-way fixed effects model to analyze whether and how overseas background executives influence corporate ESG performance, as well as how internal and external executive pay gaps facilitate this relationship. The findings reveal that executives with overseas backgrounds significantly enhance corporate ESG performance. Both internal and external executive pay gaps exhibit positive moderating effects on the relationship between overseas background executives and ESG performance. Further investigation into the heterogeneity of core relationships demonstrates that the impact of overseas background executives on ESG performance varies depending on the ownership type, size, geographic location, and industry sector of the enterprises.**

**The contributions of this study include the following: First, this paper integrates overseas background executives and corporate ESG performance into a unified analytical framework to investigate how executive characteristics enhance ESG performance. This approach broadens research on factors influencing corporate ESG performance and enriches scholarly achievements in the ESG field. Second, the study focuses on the moderating role of executive pay gaps in the relationship between overseas background executives and ESG performance. It provides theoretical guidance for designing corporate compensation systems and incentive mechanisms, while offering actionable insights for firms to refine executive compensation contracts, improve operational efficiency, and strengthen governance practices. Third, by examining heterogeneity in ownership structure, firm size, geographic location, and industry attributes, this research reveals differentiated effects of overseas background executives on corporate ESG performance. These findings provide empirical evidence to advance high-quality development in enterprises.**

**The remaining sections of this study are structured as follows. Section 2 presents the theoretical framework and hypotheses. Section 3 describes the data, variables, and empirical models. Section 4 reports the empirical analysis and results, followed by robustness checks in Section 5. Section 6 discusses the moderating effects and heterogeneity analysis. Finally, Section 7 concludes the study.**

**Eight new references are cited in the text.**

1. Zhang C, Fu P. Overseas-returned executives and their roles in firm performance: evidence from China. Asia-Pac J Account E. 2022;29(3):622-31. https://doi.org/10.1080/16081625.2020.1754257

2. Xu Z, Hou J. CEO overseas experience, dynamic capabilities and corporate digital transformation: An imprinting theory perspective. Hum Soc Sci Commun. 2024;11(1):1-14. https://doi.org/10.1057/s41599-024-03227-7

3. Qing L, Li P, Dagestani AA, Woo C, Zhong K. Does climate change exposure impact on corporate finance and energy performance? Unraveling the moderating role of CEOs’ green experience. J Clean Prod. 2024; 461:142653. https://doi.org/10.1016/j.jclepro.2024.142653

4. Qing L, Dagestani AA, Nam E, Wang C. Does firm-level exposure to climate change influence inward foreign direct investment? Revealing the moderating role of ESG performance. Corp Soc Resp Env Ma. 2024;31(6):6167-83. https://doi.org/10.1002/csr.2917

5. Liu Y, Zhang F, Zhang H. CEO foreign experience and corporate environ-mental, social, and governance (ESG) performance. Bus Strateg Environ. 2023;33(4):3331-55. https://doi. org/10.1002/bse.3647

6. Hambrick DC. Upper echelons theory: An update. Acad Manage Rev. 2007;32(2): 334-43. https://doi.org/10.5465/AMR.2007.24345254

7. Cohen S, Kadach I, Ormazabal G, Reichelstein S. Executive Compensation Tied to ESG Performance: International Evidence. J Account Res. 2023;61(3):805-53. https://doi.org/ 10.1111/1475-679x.12481

8. Meng T, Lu D, Yu D, Yahya MH, Zariyawati MA. Is executive compensation aligned with the company’s ESG objectives? Evidence from Chinese listed companies based on the PSM-DID approach. Hum Soc Sci Commun. 2024;11(1):1-15. https://doi.org/10.1057/ s41599-024-04094-y

**Comment 3:** Please elaborate on the specific content of this study in the introduction.

**Response: We sincerely thank the reviewers for their constructive suggestions. In the revised manuscript, we have addressed the reviewer’s comments and suggestions, particularly emphasizing the specific contributions and content of this study in the Introduction section. The updated text is described in the revised Introduction as follows:**

**To address the issues mentioned above, this study utilizes panel data from China's Shanghai and Shenzhen A-share listed companies spanning 2008–2022 as the research sample. We employ a two-way fixed effects model to analyze whether and how overseas background executives influence corporate ESG performance, as well as how internal and external executive pay gaps facilitate this relationship. The findings reveal that executives with overseas backgrounds significantly enhance corporate ESG performance. Both internal and external executive pay gaps exhibit positive moderating effects on the relationship between overseas background executives and ESG performance. Further investigation into the heterogeneity of core relationships demonstrates that the impact of overseas background executives on ESG performance varies depending on the ownership type, size, geographic location, and industry sector of the enterprises.**

**Comment 4:** Please state the remain structure of this study in the introduction.

**Response: We sincerely thank the reviewer for their constructive suggestions. In the revised manuscript, we have addressed the reviewer’s comments and suggestions, particularly emphasizing the specific contributions and content of this study in the Introduction section. The updated text is described in the revised Introduction as follows:**

**The remaining sections of this study are structured as follows. Section 2 presents the theoretical framework and hypotheses. Section 3 describes the data, variables, and empirical models. Section 4 reports the empirical analysis and results, followed by robustness checks in Section 5. Section 6 discusses the moderating effects and heterogeneity analysis. Finally, Section 7 concludes the study.**

**Comment 5:** The manuscript falls short in terms of theoretical development. What theories can be used as the theoretical basis for your research topic?

**Response: We sincerely thank the reviewer for their valuable suggestions. After careful consideration and internal discussion, we have adopted Upper Echelons Theory and Tournament Theory as the foundational theoretical frameworks for this study. These theories are explicitly referenced and applied in both the Introduction and the theoretical hypothesis development sections of the revised manuscript. This clarification is provided to explicitly acknowledge the adoption of these frameworks in alignment with the reviewer’s guidance.**

**Comment 6:** To better clarify the research hypotheses carried out I suggest elaborating a figure of theoretical framework in the section 2.

**Response: We sincerely appreciate the reviewer’s suggestions. As recommended, we have incorporated a theoretical framework diagram into the revised manuscript, which is now included in Part 2 of the article. The diagram is presented below:**

The theoretical model is depicted in Figure 1.

Figure 1	Theoretical model.

**Comment 7:** The measurement of all variables should be supported by references.

**Response: We sincerely thank the reviewer for their suggestions. Following a thorough review of all variables, we have supplemented the dependent variables, moderating variables, and control variables with additional references to enhance the reliability of the data and strengthen the academic foundation of the analysis. These updates are reflected in the revised manuscript.**

**For the measurement of ESG performance (the dependent variable), we draw on the methodology proposed by Qing et al. (2024).**

Qing L, Dagestani AA, Nam E, Wang C. Does firm-level exposure to climate change influence inward foreign direct investment? Revealing the moderating role of ESG performance. Corp Soc Resp Env Ma. 2024;31(6):6167-83. https://doi.org/10.1002/csr.2917

**Drawing on the methodologies of Ren et al. (2024) and Fan et al. (2024), we select the following control variables.**

1. Ren C, Lin X. The impact of performance feedback on corporate ESG performance: Mediating role of environmental strategy. Plos One. 2024;19(3):e0298471. https://doi.org/ 10.1371/journal.pone.0298471

2. Fan Z, Chen Y, Mo Y. Management myopia and corporate ESG performance. Int Rev Financ Anal. 2024; 92:103071. https://doi.org/10.1016/j.irfa.2024.103071

**Comment 8:** In the sections 4 and 6, the authors seem to "only report the results". I suggest that authors use several representative studies (2 or 3) in this area to interpret and enrich the results.

**Response: The reviewer’s suggestions have significantly enhanced the depth and practical relevance of the study. In response, we have added specific references to strengthen the theoretical and empirical foundations of the relevant sections, as detailed in Section 4 (Methodology) and Section 6 (Heterogeneity Analysis) of the revised manuscript.**

**Within Section 4, we integrate existing literature to present the regression analysis:**

**These findings broadly align with those of Liu et al. (2023), who reported a positive effect of CEO foreign experience on corporate ESG performance. Furthermore, they share similarities with Chen et al. (2023), who identified a positive association between executives’ overseas background and corporate green innovation.**

**Two references are cited in the text.**

1. Liu Y, Zhang F, Zhang H. CEO foreign experience and corporate environmental, social, and governance (ESG) performance. Bus Strateg Environ. 2023;33(4):3331-55. https://doi.org/10.1002/bse.3647

2. Chen W, Zhu Y, Wang C. Executives’ overseas background and corporate green innovation. Corp Soc Resp Env Ma. 2023;30(1):165-79. https://doi.org/10.1002/csr.2345

**Within Section 6, we added two new** **references in the part of Heterogeneity analysis.**

**The impact of overseas background executives on corporate development may vary depending on factors such as ownership type (Leng & Ben, 2025) and geographical location (Cheng & Kuang, 2024). To address these variations, we** **incorporate heterogeneity in ownership structure, firm size, region, and industry to account for potential divergences in their effects.**

**Two new references are cited in the text.**

1. Leng C, Ben F. Executives’ overseas backgrounds and green innovation in manufacturing enterprises. Financ Res Lett. 2025;72: 106585. https://doi.org/10.1016/j.frl.2024.106585

2. Cheng F, Kuang X. Effect of senior executives’ overseas experience on corporate green innovation. Plos One. 2024;19(5): e0296716. https://doi.org/10.1371/journal.pone.0296716

**Comment 9:** The authors should present the theoretical and practical implications, limitations and future research in the section conclusion.

**Response: We sincerely thank the reviewers for their constructive suggestions. Drawing on the references provided by the reviewer, we have further revised the manuscript to expand the Conclusion section, which now explicitly addresses the theoretical and practical implications, limitations, and future research directions of this study. The updated content is presented in the revised manuscript as follows.**

**7.1 Theoretical implications**

**This study enriches the existing body of knowledge in multiple dimensions.**

**First, by systematically examining the appointment of overseas background executives in corporate settings, it enhances scholarly attention to the literature on executives with international backgrounds, particularly in the context of** **Chinese listed companies.**

**Second, through empirical evidence, we demonstrate the significant impact of overseas background executives on corporate ESG performance. This contribution advances the literature on ESG outcomes and provides novel insights into Upper Echelons Theory, illustrating how executives’ experiences and values shape organizational decisions and sustainability practices.**

**Third, the study delves into the moderating roles of internal and external executive pay gaps in the relationship between overseas background executives and ESG performance—a topic rarely explored in prior research. This deepens the understanding of Tournament Theory by highlighting how incentive structures interact with leadership characteristics to drive ESG-related outcomes.**

**7.2 Practical implications**

**The findings of this study offer actionable insights for policymakers and corporate leaders.**

**First, our government should vigorously enhance ESG regulatory systems, actively promote ESG development principles, and prioritize corporate ESG performance to provide clear guidance for long-term sustainable development. At the same time, our government should also encourage enterprises across industries to recruit high-caliber overseas talent, thereby optimizing talent recruitment and team building to elevate corporate ESG performance.**

**Second, enterprises should establish long-term sustainability goals, deepen their understanding of ESG principles, integrate ESG considerations into strategic planning and decision-making systems, and emphasize balanced performance across economic, social, and environmental dimensions.**

**Third, enterprises should further refine their corporate governance structures, talent recruitment policies, and executive compensation frameworks. They must recognize and leverage the strategic value of overseas background executives in ESG-related decision-making, ensuring their expertise drives sustainable long-term corporate growth.**

**7.3 Limitations and future research**

**Despite the valuable insights provided by this study, there are still some limitations. First, while this study focuses on executives’ overseas backgrounds, it does not provide a granular analysis of these backgrounds—such as the countries of origin, duration of overseas exposure, or hierarchical authority of the executives. Additionally, other leadership characteristics (e.g., academic training and financial expertise) may shape decision-making and thereby influence corporate ESG performance. Future research should conduct detailed examinations of these dimensions to deepen understanding of how overseas experiences interact with other attributes to drive sustainability outcomes.**

**Second, while this study examines corporate ESG performance through the lens of executives with overseas backgrounds, numerous other internal factors (e.g., board diversity, innovation incentives) and external determinants (e.g., regulatory pressures, stakeholder activism) remain underexplored. Future studies could broaden the scope by systematically investigating these additional dimensions to map the drivers of ESG outcomes.**

**Third, while this study examines the impact of overseas-experienced executives on corporate ESG performance, it lacks an in-depth analysis of the underlying mechanisms. Future research should prioritize exploring the pathways through which such executives influence ESG outcomes, thereby refining and expanding the current body of knowledge.**

**Comment 10:** The language of this paper is very bad and needs help from native speakers.

**Response:** **Thanks for your suggestion. We feel sorry for our poor writings. We substantially improved the manuscript and made some changes to the manuscript. These changes will not influence the content and framework of the paper.**

**We invited Kassi Diby Francois Noel Koffi, a native speaker and foreign postdoctoral fellow at Henan University, to help edit the language of this paper. Furthermore, we collaborated with Shenyang Hai, a returned overseas Chinese, Hainan Provincial Category E High-Level Talent, PhD Supervisor at Hainan University, to enhance the language quality of this manuscript. We hope the revised manuscript will be acceptable to you.**
